# Clinical characterization and proteomic profiling of lean nonalcoholic fatty liver disease

**DOI:** 10.3389/fendo.2023.1171397

**Published:** 2023-11-16

**Authors:** Yuanye Jiang, Xiaoyu Zhuang, Jiaqi Zhang, Meng Li, Shengnan Du, Jiyun Tian, Yifu Yuan, Guang Ji, Cheng Hu

**Affiliations:** ^1^ Department of Gastroenterology, Putuo Hospital, Shanghai University of Traditional Chinese Medicine, Shanghai, China; ^2^ Experiment Center for Science and Technology, Shanghai University of Traditional Chinese Medicine, Shanghai, China; ^3^ Department of Pharmacy, Shanghai Municipal Hospital of Traditional Chinese Medicine, Shanghai University of Traditional Chinese Medicine, Shanghai, China; ^4^ Institute of Digestive Diseases, Longhua Hospital, Shanghai University of Traditional Chinese Medicine, Shanghai, China

**Keywords:** proteomic, biomarker, lipid metabolism, mass spectrometry, nonalcoholic fatty liver disease

## Abstract

**Introduction:**

Obesity has been historically associated with nonalcoholic fatty liver disease (NAFLD), but it can also occur in lean individuals. However, limited data is available on this special group. To investigate the clinical and proteomic characteristics of lean subjects with NAFLD, and to identify potential clinical variables and plasma proteins for diagnosing NAFLD in lean individuals, we collected clinical data from a large cohort of 2,236 subjects.

**Methods:**

Diagnosis of NAFLD relied on detecting pronounced hepatic steatosis through abdominal ultrasonography. Participants were categorized into four groups based on body mass index: overweight NAFLD, overweight control, lean NAFLD, and lean control. Plasma proteomic profiling was performed on samples from 20 subjects in each group. The lean NAFLD group was compared to both lean healthy and obese NAFLD groups across all data.

**Results and discussion:**

The results indicated that the lean NAFLD group exhibited intermediate metabolic profiles, falling between those of the lean healthy and overweight NAFLD groups. Proteomic profiling of plasma in lean subjects with or without NAFLD revealed 45 statistically significant changes in proteins, of which 37 showed high diagnostic value (AUC > 0.7) for lean NAFLD. These potential biomarkers primarily involved lipid metabolism, the immune and complement systems, and platelet degranulation. Furthermore, AFM, GSN, CFH, HGFAC, MMP2, and MMP9 have been previously associated with NAFLD or NAFLD-related factors such as liver damage, insulin resistance, metabolic syndromes, and extracellular homeostasis. Overall, lean individuals with NAFLD exhibit distinct clinical profiles compared to overweight individuals with NAFLD. Despite having worse metabolic profiles than their healthy counterparts, lean NAFLD patients generally experience milder systemic metabolic disturbances compared to obese NAFLD patients. Additionally, the plasma proteomic profile is significantly altered in lean NAFLD, highlighting the potential of differentially expressed proteins as valuable biomarkers or therapeutic targets for diagnosing and treating NAFLD in this population.

## 1 Introduction

Nonalcoholic fatty liver disease (NAFLD) is a condition characterized by significant lipid deposition in the liver parenchyma without history of excessive alcohol consumption. The prevalence of NAFLD has reached high levels worldwide (~25%). Around 10-30% of people with NAFLD may develop a more serious condition called nonalcoholic steatohepatitis (NASH), which is characterized by hepatic inflammation and fibrosis. In some cases, patients with NASH may even progress to cirrhosis and develop various liver-related complications due to ongoing liver injury.

NAFLD is a pathogenically complex and clinically heterogeneous disease. It is strongly linked with metabolic syndrome (MetS) components such as obesity, diabetes, dyslipidemia, and hypertension (1). Although NAFLD is more common in the obese, a small but significant subset of patients are lean, which is defined as lean or non-obese NAFLD (2, 3). The definition of lean NAFLD commonly involves ethnic-specific BMI cut-offs, such as 25 kg/m^2^ for Caucasians and 23 kg/m^2^ for Asians (4).

Non-obese NAFLD is becoming increasingly prevalent worldwide. There is a strong ethnic difference in BMI and risk of NAFLD (5). It is noteworthy that Chinese populations have similar rates of NAFLD as western populations, even at much lower BMI levels (4). Lean NAFLD patients generally experience a less severe phenotype with better histologic and biochemical profile compared to those with a higher BMI. However, they may still exhibit full range of histopathological characteristics of NASH, including steatosis, lobular inflammation, hepatocyte ballooning, and/or fibrosis (6–8). Additionally, lean NAFLD subjects are prone to similar health issues and associated diseases as their obese counterparts, such as type 2 diabetes, cardiovascular disease, and hepatocellular carcinoma. Multiple studies have demonstrated that fibrosis progression is more rapid in this population, placing them at a higher risk for the development of severe liver disease in the future. Several studies have shown that fibrosis progression is faster in this group, putting them at higher risk for severe liver disease in the future (9, 10). In fact, there is accumulating evidence to suggest that lean NAFLD might be a distinct pathophysiological entity, with approximately 47%-65% cases resulting in NASH. The underlying pathophysiology is not well understood.

The early diagnosis and intervention of NAFLD is critical due to its progressive nature. The current gold standard for NAFLD diagnosis remains histological examination of liver biopsy specimen despite it is an invasive procedure with inevitable sampling bias and interobserver variability. It is also unlikely to be widely accepted in the real life. Instead, noninvasive methods, such as biomarker panels and imaging, are widely applied for diagnosing NAFLD in clinical practice. Radiologic modalities like ultrasonography are useful screening tools widely available and relatively accurate in diagnosing of fatty liver disease, although ultrasonography is operator dependent and cannot stage liver damage progression, such as hepatic fibrosis in NAFLD patients (11). Blood-based tests for liver diseases have gained attention in recent years (12), particularly the comparison of proteomes between disease and control blood samples to discover blood biomarkers of NAFLD. Blood indicators such as alanine aminotransferase (ALT) and aspartate aminotransferase (AST) may not elevate until histological liver injury occurs. Given that ALT levels may remain normal or only intermittently elevated in many patients with NAFLD, even those with advanced fibrosis, it is urgent to find better ways to evaluate NAFLD patients. This is particularly important for identifying individuals at risk for non-obese NAFLD, who lack the typical obesity phenotype and may not seek medical attention for NAFLD diagnosis. On the other hand, despite some progress in better understanding the disease, there has been little research on lean NAFLD due to the close relationship between obesity and NAFLD.

Mass spectrometry-based proteomics technology has revolutionized our ability to explore the complex and dynamic world of proteins. This cutting-edge technology holds great potential to provide new insights into disease mechanism and biomarker discovery (13–15). In this study, we aim to identify possible clinical variables and plasma proteins that offer potential application for diagnosing NAFLD in lean subjects. Additionally, we aim to discover novel biomarkers for lean NAFLD diagnosis. We hope to identify a panel of dependable protein biomarkers, instead of relying on a single marker, which could also improve the early detection of disease among at-risk group.

## 2 Materials and methods

### 2.1 Participants

Participants were recruited from Putuo Hospital Affiliated to Shanghai University of Traditional Chinese Medicine, Shanghai, China, between March 2019 and November 2021. The **inclusion criteria** were 16-75 years of age, non-alcoholic people (alcohol intake < 210 g/week for men, <140 g/week for women), the availability of liver ultrasound data, and the availability of relevant demographic, clinical and examination information. **Exclusion criteria** were 1) laboratory or clinical evidence of autoimmune, viral, inherited causes of liver disease or of drug induced liver injury; 2) in combination with extrahepatic fibrotic diseases including systemic lupus erythematosus, rheumatic diseases, renal failure, chronic obstructive pulmonary disease, and so on;3) malignant tumors, significant cardiovascular and cerebrovascular, urinary, kidney, hematopoietic system and other severe primary diseases or complications; 4) mental illness; 5) patients with type 1 diabetes or uncontrolled type 2 diabetes mellitus (defined as HbA1c≥ 9.5%), those who have adjusted hypoglycemic drugs 2 months before enrollment, or who have experienced severe hypoglycemic events; 6) thyroid dysfunction, including hyperthyroidism, hypothyroidism, subclinical hypothyroidism, Hashimoto’s thyroiditis;

In total, 2,236 subjects meeting the criteria were recruited for this study. In line with the Guideline of prevention and treatment of nonalcoholic fatty liver disease (2018, China) formulated by the National Workshop on Fatty Liver and Alcoholic Liver Disease od Chinese Medical Association and Fatty Liver Disease Expert Committee of Chinese Medical Doctor Association (16), in the absence of other causes of fatty liver disease, NAFLD was diagnosed based on the detection of significant hepatic steatosis on abdominal ultrasonography. To minimize the subjective influence of different doctors on the results, each examination of included patients in this study was conducted by two ultrasound physicians. The protocol received approval from the Medical Ethics Committee. All participants have signed informed consent.

Ethnic-specific BMI cut-offs of 25 kg/m2 for Caucasians and 23 kg/m2 for Asians are typically used to define the “lean” population. For our study, only Chinese participants were included, and we used the Asian-specific BMI criteria to classify individuals as “lean” (BMI < 23) or “overweight” (BMI ≥ 23). Controls were healthy volunteers with no signs of liver disease or other chronic diseases. Participants were divided into two cohorts. The overweight cohort included overweight NAFLD group (n=1,100) and overweight control group (n=312), the lean cohort consisted of lean NAFLD group (n=403) and lean control group (n=421). Samples of 20 subjects in each group were used for plasma proteomic profiling.

### 2.2 Clinical and laboratory assessment

Venous blood was collected after overnight fasting for a minimum of 12 h. Participants underwent a standard physical examination that included blood pressure, height, weight, and waist circumference and hip circumference. Body mass index (BMI) was measured as the body weight in kilograms divided by the square of the height of the body in meters (kg/m^2^). Detailed medical histories were obtained via questionnaire. Laboratory investigations included measurement of routine blood examination, fasting plasma glucose, lipid panel, liver biochemistry, and renal biochemistry, thyroid function tests were carried out on an automated chemistry analyzer (Hitachi 7600d-210, Japan).

The FIB-4 index was employed to assess the likelihood of significant liver fibrosis in participants. A higher FIB-4 score indicates a greater likelihood of fibrosis. FIB-4 scores are interpreted as below: scores below 1.30 indicate a low risk of significant fibrosis; scores between 1.30 and 2.67 indicate an indeterminate risk of significant fibrosis, requiring further evaluation; scores equal to or above 2.67 indicate a high risk of significant fibrosis (17–19)

### 2.3 Peptides preparation for MS analysis (high abundant protein depletion, protein extraction and digestion)

Removal of high abundant plasma proteins was performed using a High-Select™ Top14 Abundant Protein Depletion Spin Column (Thermo Fisher Scientific) in accordance with the manufacturer’s instructions. In brief, 10 μL of plasma sample was loaded onto the column and incubated at room temperature with gentle end-over-end mixing for 10 min. The filtrate was then collected by centrifuging at 1,000 g for 2 min. The resulting depleted protein sample was precipitated with acetone at -20°C for 2 h, followed by dissolution in 6 M guanidine hydrochloride and 50 mM ammonium bicarbonate (NH_4_HCO_3_, pH 8.0). The protein concentration was determined using BCA protein assay.

Dithiothreitol (final concentration of 20 mM) was added to the solution and incubate at 57°C for 30 min to reduce disulfide bonds, then iodoacetamide (final concentration of 80 mM) for alkylation of the free thiol group was added and incubated at room temperature for 30 min in the dark. To exchange the buffer and digest the protein, we employed filter-aided sample preparation (FASP) method developed by Wisniewski et al. (20) Briefly, proteins were loaded in 10 kDa centrifugal filter tubes (Merck) and washed with 50 mM NH_4_HCO_3_ for three times. Afterwards, samples were treated with sequence-level trypsin (Promega, Madison, MI) at an enzyme substrate ratio of 1:50 for 16 h at 37°C in 50 mM NH_4_HCO_3_. The peptides were eluted by centrifugation and desalted using a MonoSpin C18 column (GL Science, Tokyo, Japan).

### 2.4 LC-MS/MS proteomic analysis

Peptide samples were analyzed on an Easy-nLC 1000 system (Thermo Fisher Scientific) coupled with an Orbitrap Fusion Lumos mass spectrometer (Thermo Fisher Scientific). Separation of the peptide mixture was achieved using a PepMap C18 column (2μm, 75μm*250mm) at a flow rate of 300 nL/min over a 120 min gradient (mobile phase A was 0.1% formic acid in water; mobile phase B was 0.1% formic acid in acetonitrile). The analytical column temperature was set at 50°C during the experiments. The elution gradient was as follows: 0–2 min, 2 to 8% B; 2–82 min, 8 to 28% B; 82–102 min, 28 to 32% B; 102–120 min, 32 to 95% B.

Mass spectrometry was operated under a data-dependent acquisition (DDA) mode. The spray voltage was set at 2,200 V in positive ion mode and the ion transfer tube temperature was set to 275°C. For the MS1 full scan, ions with *m/z* ranging from 350 to 1,800 were acquired by Orbitrap mass analyzer at a resolution of 120,000. The automatic gain control (AGC) target was set as 1e6 and the maximum ion injection time was 50 ms. For the MS2 acquisition, a top-speed mode was employed with a duty cycle time of 3 s. Precursor ions were selected and fragmented with higher energy collision dissociation (HCD) of 30%. The resulting fragment ions were analyzed by Orbitrap mass analyzer, with the MS2 scan resolution set to 15,000 and an isolation window of 1.6 m/z. The AGC and maximal ion injection time for MS2 were set at 1e5 and 22 ms, respectively. A dynamic exclusion time of 60 s was applied.

### 2.5 MS database searching and differential protein analysis

MS raw data were processed using Proteome Discoverer 2.4 and searched against Swissprot human proteome database (released on June 20, 2021). Trypsin was set as the protease. The maximum number of missing cleavage site was set to 2 with a minimum peptide length of 7. The false discovery rates (FDR) of peptide, protein and site were all < 0.01. Carbamidomethyl (C) was considered as a fixed modification, and oxidized methionine, protein N-term acetylation, asparagine and glutamine deamidation were set as variable modifications.

A label-free quantification algorithm was used for protein quantitation. The protein abundance in each sample was determined as the sum of all normalized peptide areas for a given protein. For statistical analysis, proteins with valid values in at least 70% of the samples in one experimental group were further considered. Proteins with altered expression in NAFLD compared to healthy controls were identified by a two-sided t-test (P value < 0.05) and were defined as upregulated or downregulated if the N/H ratio was greater than 1.20 or less than 0.833, respectively. The top Gene Ontology (GO)/annotation terms were enriched using Cytoscape plug-in ClueGO and DAVID Bioinformatics Resource 6.8.

### 2.6 Statistical analysis

Statistical analysis was carried out using the statistical software SPSS 25.0 (SPSS Inc. Chicago, United States). Chicago, United States). The Pearson χ2 test was applied to categorical variables based on the sample size and theoretical frequency, the continuous correction formula of chi-square test or Fisher’s exact probability method was used if necessary. Continuous normally distributed variables were compared between groups using Student’s T test, expressed as mean ± standard deviation (SD), t-test for homogeneity of variance, and t’ test for unequal variance. Continuous variables that were not normally distributed were compared between groups using the Mann-Whitney U test and reported as M (P25, P75). A p value less than 0.05 was considered statistically significant in all cases. To assess the diagnostic power of each potential biomarker in NAFLD, we used the area under the receiver operating characteristic (ROC) (AUC) curve.

## 3 Results

### 3.1 Clinical and biochemical characteristics

**Figure 1:**
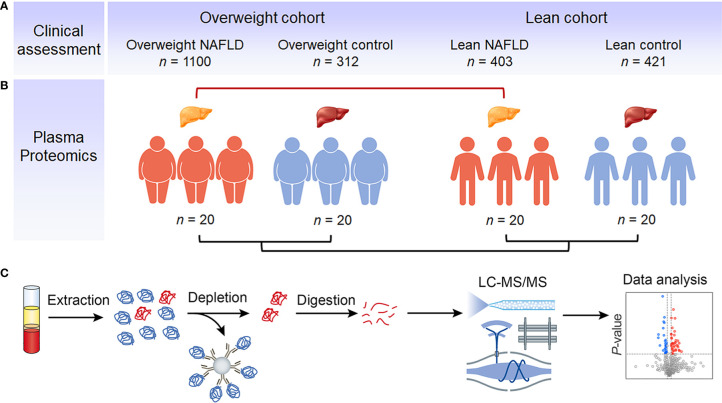
Study outline and proteomic workflow. Using BMI of 23 as the cut-off value, eligible NAFLD patients and healthy individuals were divided into overweight NAFLD group, overweight control group, lean NAFLD group and lean control group. The clinical data were collected from a large cohort of 2,236 subjects, with the number of subjects in each group indicated **(A)**. Samples of 20 subjects in each group were used for plasma proteomic profiling **(B)**. Fasting plasma was collected and analyzed using a MS-based proteomics strategy, including protein extraction, high abundant protein depletion, protein digestion, LC-MS/MS analysis, database search, and further computational analysis **(C)**.

We collected clinical data from a large cohort of 2,236 subjects and used samples from 20 subjects in each group for plasma proteomic profiling. **Figure 1** illustrates the overall study design. To exclude the influence of BMI, we initially compared the clinical characteristics and proteome profiles of subjects within each cohort, i.e., overweight NAFLD vs. overweight control, lean NAFLD vs. lean control. To better understand the differences in the pathogenesis of NAFLD between lean and overweight individuals, we then compared the data of lean NAFLD patients to that of overweight NAFLD patients. The demographic, clinical and biochemical characteristics are shown in **Table 1**.

**Table 1:** Demographic, clinical, anthropometrical and laboratory characteristics of the study population.

Variables	Lean NAFLD (n=403)	Lean control (n=421)	Overweight NAFLD (n=1100)	Overweight control (n=312)		P-value	
					LN-LC	ON-OC	LN-ON
Demographic data & physical characteristics							
Gender (M/F)	135/268	78/343	636/464	145/167	<0.001	<0.001	<0.001
Age (year)	48.00(37.00,58.00)	42.00(31.00,51.00)	53.00(44.00,65.00)	50.00(39.25,58.75)	<0.001	<0.001	<0.001
Height (cm)	163.00(158.00,172.00)	162.00(157.00,167.00)	167.00(160.00,173.00)	164.00(157.00,172.00)	<0.001	0.009	<0.001
Weight (kg)	58.00(54.00,64.00)	54.00(51.00,59.00)	74.00(66.00,82.00)	68.00(61.25,75.00)	<0.001	<0.001	<0.001
BMI (kg/m^2^)	22.10(21.00,22.80)	20.90(19.80,21.90)	26.60(25.10,28.50)	24.71(23.84,26.23)	<0.001	<0.001	<0.001
FIB4 index	0.96(0.65,1.39)	0.90(0.57,1.31)	0.93(0.30,1.51)	0.99(0.55,1.44)	<0.05	0.370	0.189
Blood routine examination							
FBG (mmol/L)	4.90(4.60,5.40)	4.80(4.50,5.10)	5.30(4.80,5.90)	5.00(4.70,5.40)	<0.001	<0.001	<0.001
WBC (×109/L)	6.10(5.10,7.15)	5.70(4.80,6.70)	6.50(5.60,7.50)	6.00(5.20,7.00)	<0.001	<0.001	<0.001
RBC (%)	4.53(4.26,4.87)	4.34(4.10,4.64)	4.77(4.47,5.09)	4.61(4.33,4.89)	<0.001	<0.001	<0.001
Haemoglobin (g/L)	137.00(126.00,148.00)	130.00(123.00,140.00)	145.00(134.00,155.00)	140.00(128.25,150.00)	<0.001	<0.001	<0.001
Haematocrit (%)	40.80(38.00,43.70)	38.80(36.70,41.40)	42.90(40.20,45.50)	41.20(38.40,44.20)	<0.001	<0.001	<0.001
MCV (fL)	89.40(87.05,92.10)	89.60(87.30,92.30)	90.00(87.30,92.20)	89.70(87.20,92.60)	0.513	0.801	0.126
MCH (pg)	30.20(29.10,31.10)	30.30(29.20,31.10)	30.50(29.50,31.40)	30.50(29.60,31.40)	0.576	0.824	<0.001
MCHC (g/L)	336.00(330.00,342.00)	335.00(329.00,342.00)	337.00(331.00,344.00)	338.00(331.00,343.00)	0.294	0.533	0.008
Platelet (×109/L)	239.00(206.00,285.00)	240.00(206.00,281.00)	243.00(206.00,284.00)	228.00(195.00,270.00)	0.894	<0.001	0.629
RDW (×1012/L)	12.40(12.10,13.10)	12.50(12.00,13.10)	12.50(12.10,13.00)	12.50(12.10,13.00)	0.768	0.758	0.881
MPV (fL)	10.60(10.00,11.20)	10.60(10.10,11.20)	10.42(9.90,11.20)	10.60(10.00,11.20)	0.549	0.031	0.040
Neutrophil (%)	55.70(50.60,61.60)	54.40(49.10,60.40)	55.70(50.30,61.00)	55.90(50.00,60.70)	0.031	0.837	0.770
Lymphocyte (%)	34.30(28.65,39.60)	35.40(30.10,40.90)	33.80(28.60,38.80)	33.50(29.00,38.90)	0.025	0.901	0.315
Monocyte (%)	6.80(6.00,8.10)	6.90(6.00,7.80)	7.00(6.10,8.30)	7.30(6.20,8.50)	0.797	0.142	0.070
Eosinophil (%)	1.70(1.10,2.80)	1.70(1.00,2.80)	2.10(1.30,3.30)	1.80(1.20,3.30)	0.970	0.068	<0.001
Basophils (%)	0.50(0.30,0.70)	0.50(0.30,0.70)	0.50(0.40,0.70)	0.50(0.40,0.70)	0.044	0.838	<0.001
Renal function tests							
BUN (mmol/L)	5.10(4.30,5.95)	4.80(4.10,5.60)	5.30(4.50,6.20)	5.10(4.30,6.00)	0.004	0.008	<0.001
Creatinine (μmol/L)	62.00(55.00,73.00)	60.00(55.00,68.00)	70.00(59.00,81.00)	68.00(57.00,79.00)	0.111	0.079	<0.001
Uric acid (μmol/L)	317.00(271.00,374.50)	288.00(251.00,331.00)	380.00(322.00,447.00)	334.00(276.00,406.00)	<0.001	<0.001	<0.001
GFR (mL/min)	101.61(89.02,112.46)	100.39(87.20,117.95)	99.96(87.47,113.70)	93.17(76.56,109.40)	0.815	0.108	0.879
Liver function tests							
TB (μmol/L)	13.00(10.00,18.00)	13.00(10.25,16.75)	14.00(11.00,18.00)	14.00(10.00,18.00)	0.722	0.426	0.317
DB (μmol/L)	2.45(1.80,3.30)	2.60(1.90,3.20)	2.50(1.90,3.30)	2.50(1.78,3.20)	0.506	0.363	0.172
AKP (U/L)	68.00(57.00,86.00)	62.00(51.00,74.75)	78.00(65.00,92.00)	70.00(56.00,82.00)	<0.001	<0.001	<0.001
TP (g/L)	74.00(70.00,76.00)	74.00(71.00,76.00)	74.00(71.00,76.00)	72.00(70.00,75.00)	0.795	0.001	0.545
Albumin (g/L)	43.00(40.00,45.50)	45.00(43.00,46.00)	44.00(43.00,46.00)	44.00(42.00,45.00)	<0.001	0.042	<0.001
γ-GT (U/L)	21.00(14.00,32.00)	15.00(12.00,21.00)	31.00(20.75,47.00)	21.00(15.00,36.00)	<0.001	<0.001	<0.001
CHE (U/L)	8726.00(7789.25,9824.00)	7802.00(6935.00,8712.00)	9123.00(8311.00,10124.00)	8418.00(7399.25,9296.00)	<0.001	<0.001	0.003
ALT (U/L)	13.00(9.00,19.00)	10.00(7.00,13.00)	18.00(13.00,26.00)	14.00(10.00,18.00)	<0.001	<0.001	<0.001
AST (U/L)	21.00(17.00,25.00)	20.00(17.00,23.00)	24.00(19.00,29.00)	21.00(17.75,26.00)	<0.001	<0.001	<0.001
TBA (μmol/L)	3.00(2.00,6.00)	3.00(2.00,4.00)	3.00(2.00,5.00)	3.00(2.00,5.00)	0.072	0.312	0.723
Lipid panel							
HDL-C (mmol/L)	1.27(1.08,1.47)	1.44(1.28,1.66)	1.13(1.00,1.30)	1.25(1.07,1.48)	<0.001	<0.001	<0.001
LDL-C (mmol/L)	3.34(2.75,3.87)	3.11(2.70,3.63)	3.50(2.99,3.97)	3.30(2.82, 3.89)	0.007	0.006	<0.001
TC (mmol/L)	5.11(4.47,5.91)	4.91(4.40,5.68)	5.23(4.63,5.93)	5.07(4.34,5.86)	0.008	0.004	0.102
TG (mmol/L)	1.35(0.90,1.99)	0.91(0.71,1.27)	1.70(1.24,2.43)	1.23(0.92,1.69)	<0.001	<0.001	<0.001
ApoA1 (g/L)	1.39(1.26,1.63)	1.52(1.34,1.75)	1.32(1.18,1.48)	1.38(1.23,1.60)	<0.001	<0.001	<0.001
ApoB (g/L)	0.91(0.76,1.09)	0.81(0.69,0.95)	1.00(0.84,1.16)	0.91(0.78,1.06)	<0.001	<0.001	<0.001
Lp(a) (mg/L)	60.00(35.00,124.00)	69.00(38.00,142.25)	59.50(34.00,132.75)	78.00(40.00,185.75)	0.362	0.010	0.910
Thyroid function tests							
TgAb (IU/mL)	1.74(1.00,15.30)	1.89(1.00,25.00)	1.32(1.00,4.48)	1.14(1.00,3.09)	0.242	0.349	0.052
TPOAb (IU/mL)	1.00(1.00,3.70)	1.00(1.00,3.99)	1.00(1.00,2.21)	1.30(1.00,5.70)	0.713	0.008	0.085
Tg (ng/mL)	5.41(3.39,8.24)	5.20(3.02,8.08)	5.16(3.14,8.46)	5.39(3.05,8.52)	0.166	0.976	0.328
TRAb (IU/L)	0.10(0.10,0.10)	0.10(0.10,0.35)	0.10(0.10,0.10)	0.34(0.10,0.66)	0.016	<0.001	0.220
T3(nmol/L)	1.90(1.69,2.10)	1.81(1.66,1.98)	1.95(1.75,2.20)	1.85(1.69,2.04)	0.019	0.006	0.063
T4(nmol/L)	101.00(92.70,113.00)	99.40(91.55,109.00)	101.00(91.70,114.00)	102.00(88.75,112.00)	0.280	0.517	0.629
FT3 (pmol/L)	5.05(4.68,5.41)	4.80(4.61,5.28)	5.04(4.71,5.43)	5.03(4.70,5.42)	0.041	0.875	0.788
FT4 (pmol/L)	11.10(10.20,12.00)	10.90(10.00,11.85)	10.70(10.00,11.70)	11.20(10.20,12.60)	0.274	0.003	0.035
TSH (μIU/L)	2.90(1.99,3.81)	2.64(1.86,3.81)	2.53(1.91,3.34)	2.11(1.64,3.12)	0.155	0.005	0.006

All variables are expressed as median (interquartile range) unless otherwise indicated. P -value assessed by Mann–Whitney U-test.

LN, lean NAFLD; LC, lean control; ON, overweight NAFLD; OH, overweight healthy; FBG, fasting blood glucose; WBC, white blood cells; RBC, red blood cells; MCV, mean corpuscular volume; MCH, mean corpuscular haemoglobin; MCHC, mean corpuscular haemoglobin concentration; RDW, red blood cell distribution width; MPV, mean platelet volume; BUN, blood urea nitrogen; GFR, glomercular filtration rate; TB, total Bilirubin; DB, direct bilirubin; AKP, alkaline phosphatase; TP, total protein; γ-GT, γ-glutamyl transferase; CHE, cholinesterase; ALT, alanine aminotransferase; AST, aspartate aminotransferase; TBA, total bile acid; HDL-C, high density lipoprotein cholesterol; LDL-C, low density lipoprotein cholesterol; TC, total cholesterol; TG, triglyceride; ApoA1, apolipoprotein A1; ApoB, apolipoprotein B; Lp(a), lipoprotein(a); TgAb, thyroglobulin antibody; TPOAb, thyroid peroxidase antibody; TgAb, thyroglobulin antibody; Tg, thyroglobulin; TRAb, thyrotropin receptor antibody; T3, triiodothyronine; T4, thyroxine; FT3, free T3; FT4, free T4; TSH, thyroid stimulating hormone.

The results show that middle-aged subjects have a higher prevalence of NAFLD. The overweight NAFLD group included 636 males (57.8%) and 464 females (42.2%), while the lean NAFLD group had a higher proportion of females (66.5%). Both the lean and overweight NAFLD groups had slightly higher BMIs than their respective healthy counterparts. Although the mean aminotransferase levels in lean NAFLD fall within the normal range, NAFLD patients in both lean and overweight cohorts exhibited elevated levels of ALT and AST, as well as AKP, γ-GT and CHE compared to their healthy counterparts.

Significant differences in almost all blood lipid indices, including HDL, LDL, TC, TG, APOB, and APOA1, were observed in both cohorts. While this study did not include subjects with hyperlipidemia or hypertriglyceridemia, both the overweight and lean NAFLD groups showed significantly higher levels of serum LDL, TC, TG, APOB, and lower levels of HDL and APOA1 than their healthy counterparts. These findings emphasize that a disorder of lipid metabolism can be present and may contribute to fatty liver disease in individuals with a normal weight.

As for the complete blood count test, significant higher levels of HGB, HCT, RBC and WBC were observed in both lean and overweight NAFLD groups when compared to their respective control groups. The overweight NAFLD patients exhibited the highest levels of HGB, HCT, RBC, and WBC. In addition, NAFLD patients also had significant raised fasting blood glucose, indicating a worse metabolic profile. According to research, an elevated platelet count might be linked to NASH (21), but this correlation was only found among overweight individuals with NAFLD compared to overweight controls. Lean NAFLD patients exhibited even significantly lower platelet count compared to their healthy counterparts.

The thyroid hormones are essential regulators of metabolism including lipid metabolism in the liver. Our findings in the overweight cohort were consistent with previous reports, showing a direct relationship between NAFLD and decreasing levels of FT4 (22). However, there was no significant difference in FT4 levels between the two lean groups. Both overweight and lean NAFLD patients showed increased levels of TSH, which is positively linearly associated with NAFLD risk, even within the euthyroid reference range (23). Notably, we found that lean NAFLD subjects had significantly higher TSH levels than overweight NAFLD subjects (P<0.01).Numerous observational studies suggest that individuals with NAFLD have a significantly higher incidence of CKD compared to those without NAFLD (24–26). Uric acid (UA) and blood urea nitrogen (BUN) levels were notably elevated in both lean and overweight NAFLD patients compared to their matched healthy controls, with the highest levels of these indicators observed in overweight NAFLD. While creatinine has been reported as a factor associated with NAFLD in several studies (8) (27), we did not observe statistically significant differences in creatinine levels between NAFLD patients and controls in both overweight and lean cohorts.

### 3.2 Plasma proteome profiling

#### 3.2.1 Overview

Label-free quantitative proteomics was conducted on plasma samples from 40 NAFLD patients and 40 healthy controls. The clinical characteristics of these subjects are shown in **Table 1**. After depletion of high-abundant blood proteins, a total of 959 proteins were identified, with 677 of these proteins being identified with ≥ 2 unique peptides. We quantified on average 621 proteins per individual. The dataset was filtered to ensure a 70% data completeness in at least one experimental group. Proteins that show a significant change >20% (with fold changes of > 1.20 or < 0.83) between any two groups (p<0.05) were considered as differentially expressed proteins (DEPs).

**Figure 2:**
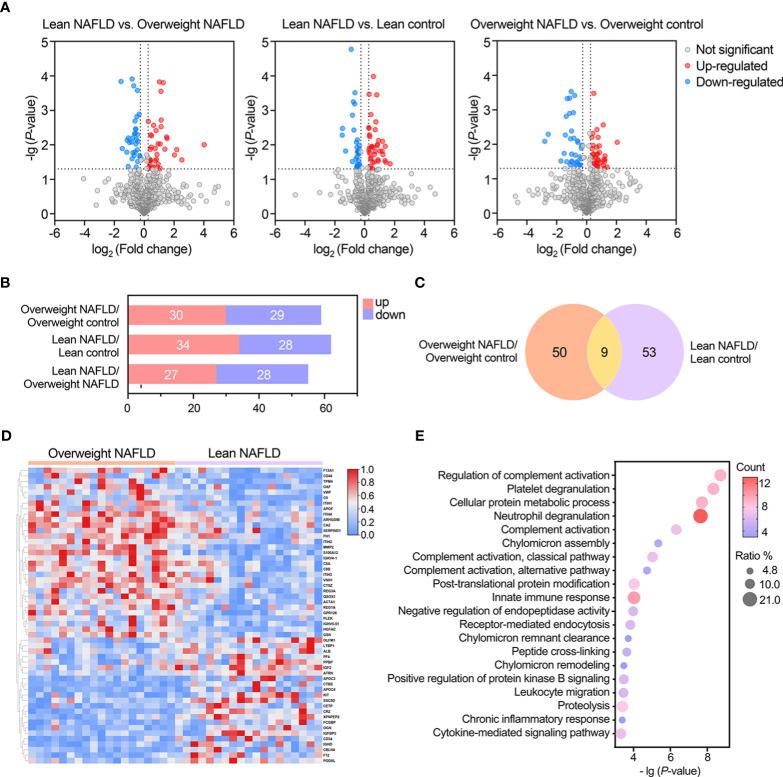
Statistical and bioinformatic analysis of the identified proteins reveled proteomic alterations associated with lean NAFLD and overweight NAFLD. **(A)** Volcano plots of identified proteins in the comparisons between lean NAFLD group vs. lean control group, overweight NAFLD group vs. overweight control group, and lean NAFLD group vs. overweight control group (cut-off value of fold-change >1.2; P-value (t-test) <0.05). **(B)** Bar chart shows the numbers of significantly up- and down-regulated proteins in the between- group comparisons. **(C)** Venn diagram shows the overlap of significantly differently expressed proteins found in lean and overweight cohort. **(D)** Hierarchical clustering heatmap of DEPs between lean NAFLD group and overweight control group. **(E)** GO enrichment of differentially expressed proteins associated with lean NAFLD in term of biological process.

We first compared the proteome profiles of lean and overweight NAFLD patients and found that 54 proteins were significantly different between the two groups (**Table S2**). This suggests that lean NAFLD might be a distinct pathophysiological entity. Volcano plots were used to illustrate variations in the proteome profile, showing the ratio of mean protein concentrations of lean NAFLD to overweight NAFLD subjects (**Figure 2A**). In addition, a heat map was generated by hierarchically clustering the variables that effectively discriminating between the two groups with fold change > 1.20 or < 0.83 (**Figure 2D**).

When comparing the plasma proteome profiles of lean NAFLD to that of lean healthy controls, we identified 62 proteins that differ significantly between the two groups, with 34 proteins were upregulated and 28 were downregulated (**Table 2** and **Figures 2A, B**). To validate the biological relevance of this dataset, pathway annotation was conducted using the ClueGO plug-in in Cytoscape and DAVID Bioinformatic Resource. The results suggest that these 62 candidate proteins are mainly involved in biological processes such as complement activation, platelet degranulation, neutrophil degranulation, cell adhesion, and immune and inflammatory response (**Figure 2E**).

**Table 2:** List of proteins found to be significantly differentially abundant between lean NAFLD patients and lean controls, with efficiency comparison of diagnostic indicators.

Gene Symbol	Protein	P value (LN vs. LC)	FC (LN/LC)	AUC	CI (95%)	
					lower	upper
APOF	Apolipoprotein F	0.0000	0.47	0.959	0.903	1.000
C9	Complement component C9	0.0000	0.45	0.944	0.873	1.000
CA2	Carbonic anhydrase 2	0.0000	0.56	0.895	0.797	0.992
C8B	Complement component C8 beta chain	0.0001	0.67	0.880	0.765	0.995
GSN	Gelsolin	0.0000	0.59	0.871	0.759	0.983
APOH	Beta-2-glycoprotein 1	0.0003	1.31	0.863	0.744	0.981
VWF	von Willebrand factor	0.0001	0.68	0.857	0.736	0.978
ATRN	Attractin	0.0001	1.28	0.854	0.706	1.000
ITIH3	Inter-alpha-trypsin inhibitor heavy chain H3	0.0001	0.61	0.845	0.718	0.972
CD163	Scavenger receptor cysteine-rich type 1 protein M130	0.0001	1.53	0.842	0.712	0.972
CFH	Complement factor H	0.0003	1.26	0.836	0.711	0.962
PON3	Serum paraoxonase/lactonase 3	0.0001	0.53	0.836	0.706	0.967
ITIH1	Inter-alpha-trypsin inhibitor heavy chain H1	0.0003	0.78	0.827	0.692	0.963
MMP2	72 kDa type IV collagenase	0.0006	0.52	0.825	0.687	0.963
C4BPA	C4b-binding protein alpha chain	0.0005	1.23	0.822	0.689	0.955
ARHGDIB	Rho GDP-dissociation inhibitor 2	0.0003	0.61	0.819	0.684	0.954
LTF	Lactotransferrin	0.0010	1.71	0.810	0.660	0.959
VNN1	Pantetheinase	0.0012	0.64	0.810	0.666	0.954
QSOX1	Sulfhydryl oxidase 1	0.0016	0.62	0.804	0.657	0.952
CTSZ	Cathepsin Z	0.0036	0.59	0.789	0.643	0.936
ITIH2	Inter-alpha-trypsin inhibitor heavy chain H2	0.0018	0.68	0.787	0.640	0.933
PTPRJ	Receptor-type tyrosine-protein phosphatase eta	0.0066	1.28	0.787	0.637	0.936
F13A1	Coagulation factor XIII A chain	0.0075	0.73	0.781	0.629	0.933
SSC5D	Soluble scavenger receptor cysteine-rich domain-containing protein SSC5D	0.0009	1.64	0.781	0.630	0.931
THBS1	Thrombospondin-1	0.0011	0.69	0.781	0.627	0.935
ICAM2	Intercellular adhesion molecule 2	0.0064	1.53	0.769	0.610	0.928
APOB	Apolipoprotein B-100	0.0044	0.69	0.763	0.610	0.916
BTD	Biotinidase	0.0034	1.32	0.757	0.604	0.911
CAP1	Adenylyl cyclase-associated protein 1	0.0015	0.33	0.754	0.589	0.920
CNTN1	Contactin-1	0.0049	0.68	0.754	0.592	0.917
IGFBP3	Insulin-like growth factor-binding protein 3	0.0081	1.31	0.751	0.584	0.919
AFM	Afamin	0.0074	1.24	0.749	0.588	0.909
HGFAC	Hepatocyte growth factor activator	0.0185	0.77	0.746	0.586	0.905
OLFM1	Noelin	0.0056	1.41	0.743	0.582	0.904
SPP2	Secreted phosphoprotein 24	0.0306	0.67	0.725	0.552	0.898
SERPINA7	Thyroxine-binding globulin	0.0270	0.79	0.722	0.558	0.886
GOLM1	Golgi membrane protein 1	0.0249	0.67	0.716	0.548	0.884
IGHV3-7	Immunoglobulin heavy variable 3-7	0.0121	0.72	0.716	0.551	0.882
SERPINF1	Pigment epithelium-derived facto	0.0065	1.43	0.711	0.544	0.877
S100A12	Protein S100-A12	0.0007	0.34			
TXN	Thioredoxin	0.0009	1.63			
FLNA	Filamin-A	0.0013	1.47			
LCN2	Neutrophil gelatinase-associated lipocalin	0.0027	1.71			
IGKV3D-20	Immunoglobulin kappa variable 3D-20	0.0036	1.56			
IGKV2-30	Immunoglobulin kappa variable 2-30	0.0039	0.51			
ANPEP	Aminopeptidase N	0.0067	1.41			
FAM3C	Protein FAM3C	0.0077	3.54			
APOC3	Apolipoprotein C-III	0.0101	1.83			
CFI	Complement factor I	0.0119	1.24			
ENO1	Alpha-enolase	0.0119	1.72			
P4HB	Protein disulfide-isomerase	0.0143	2.10			
IGFBP7	Insulin-like growth factor-binding protein 7	0.0180	2.17			
APOC2	Apolipoprotein C-II	0.0199	2.20			
KIT	Mast/stem cell growth factor receptor Kit	0.0202	1.79			
MMP9	Matrix metalloproteinase-9	0.0209	1.63			
ITGB1	Integrin beta-1	0.0226	0.68			
ENG	Endoglin	0.0267	1.89			
CFP	Properdin	0.0310	1.32			
FCGBP	IgGFc-binding protein	0.0335	1.47			
KRT5	Keratin, type II cytoskeletal 5	0.0363	1.52			
S100A8	Protein S100-A8	0.0444	2.59			
PODXL	Podocalyxin	0.0483	1.27			

LN, lean NAFLD; LC, lean control; ON, overweight NAFLD; OC, overweight control; FC, fold change; AUC, area under the receiver operating characteristic (ROC) curve.

In the comparison between overweight NAFLD and overweight healthy controls, significant changes were observed in the levels of 59 proteins, of which 30 and 29 were up- and down-regulated, respectively (**Table S1** and **Figures 2A, B**). GO enrichment of DEPs associated with overweight NAFLD in term of biological process is shown in **Figure S1**. As shown in Venn diagram (**Figure 2C**), among the DEPs identified in the overweight cohort, 9 proteins also differed significantly in abundance by NAFLD status within the lean cohort. Of note, these 9 overlapped DEPs displayed similar up-/down-regulated trend (APOF, GOLM1, IGHV3-7, MMP9, THBS1, S100A8, S100A12, TXN, LCN2) in both comparisons. Moreover, in the comparison of lean NAFLD and overweight NAFLD with their corresponding healthy controls, most of the DEPs were annotated as liver-specific or liver-enriched, indicating dysregulated hepatic protein synthesis and secretion in patients with NAFLD.

#### 3.2.2 Potential biomarkers for diagnosing lean NAFLD

As mentioned above, 59 and 62 DEPs were identified in the overweight and lean cohorts, respectively. We then assessed the diagnostic power of each candidate biomarkers for NAFLD within each cohort by identifying classifiers/components with an area under the ROC curve (AUC) > 0.7. **Tables 2**, **S1** shows a total of 39 and 43 proteins fulfill the criterion and could serve as biomarkers for diagnosing NAFLD among lean and overweight cohorts, respectively. Among these, APOF, GOLM1, IGHV3-7 and THBS1 were found in both cohorts.

**Figure 3:**
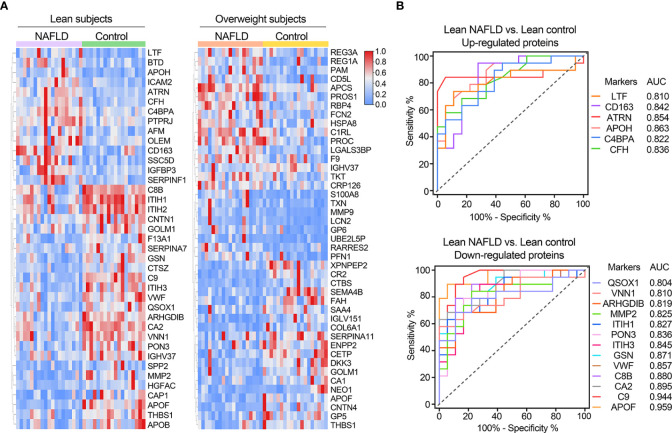
**(A)** Clustering heatmap of signature proteins that were differentially expressed in NAFLD and control groups (proteins with AUCs above 0.7 are shown). Clustering heatmap of proteins significantly differentially expressed in NAFLD and control groups in lean and obese cohorts, respectively (proteins with AUCs above 0.7 are shown). **(B)** Receiver operating characteristic (ROC) curves for potential diagnostic markers of lean NAFLD with AUC values above 0.8.

**Table 2** provides a detailed summary of the AUCs, as well as the lower and upper limit of the 95% CI, sensitivities and specificities of the identified plasma proteins Hierarchical clustering was used to group all candidate biomarkers by similarities in their expression patterns (mean log2 intensities) among both cohorts (**Figure 3A**). ROC curves for proteins with high diagnostic power for NAFLD (AUC >0.8) in both lean and overweight individuals were also shown (**Figures 3B**, **S2**). After excluded the overlapped proteins which are statistically significantly different in both cohorts, a panel of 35 proteins that exclusively identified in lean cohort were considered as specific biomarkers for diagnosing lean NAFLD.

#### 3.2.3 Plasma proteins highly associated with NAFLD observed in overweight cohort

Of the 55 proteins that exhibited significant changes in the comparison of overweight NAFLD with overweight healthy controls, 43 proteins reached an AUC > 0.7 (**Table S1**). As expected, some of these proteins have already been linked to NAFLD, such as RBP4, CETP, APCS, CD5L, and MMP9.

Retinol-binding protein 4 (RBP4) is synthesized in the liver that responsible for the transport of retinol to peripheral tissues. Available studies have described the correlation between RBP4 and NAFLD demonstrated that elevated RBP4 levels may contribute to the development of this condition (28–30). Our findings are consistent with these reports, as we observed significantly higher RBP4 levels in overweight NAFLD patients compared to the healthy control group. By moving HDL-associated cholesterol to other lipoproteins, cholesteryl ester transfer protein (CETP) can affect the transport of peripheral tissue cholesterol, ultimately altering the levels of LDL and HDL cholesterol. We observed significantly decreased plasma CETP level in the overweight NAFLD group compared to healthy controls may support the reported finding that CETP plays a protective role in lipid and lipoprotein metabolism in obesity (31).Acute phase proteins, which play a crucial role in the innate defense of the liver, are immediately involved in hepatocyte injury. The differential expression of two acute phase proteins, serum amyloid P-component (APCS) and CD5 antigen-like protein (CD5L), between two overweight groups, suggests their potential as predictive markers for NAFLD in obese patients, as they regulate the innate and adaptive immune systems (15, 32).

#### 3.2.4 Dysregulated lipid metabolism in lean NAFLD

Our proteomic data uncovered dysregulation of multiple apolipoproteins, including APOB, APOC2, APOC3, APOF, and APOH, in lean individuals with NAFLD compared to healthy lean individuals.

We found that apolipoprotein F (APOF) and apolipoprotein H (APOH) had significant diagnostic value for lean NAFLD, with AUC values of 0.959 and 0.863, respectively. Current data support that APOF preferentially blocks CETP activity when it is bound to LDL, thus reducing the flow of HDL-derived cholesteryl ester derived from HDL through this pathway (33). We found down-regulated plasma levels of APOF in both lean and overweight NAFLD groups compared to their matched healthy subjects, with the lowest level observed in lean NAFLD patients. Along this line, it is not surprising that the lean NAFLD group exhibited significantly higher levels of CETP than the overweight NAFLD group. Additionally, elevated levels of APOH, APOC2, and APOC3 have been linked to clinically apparent arteriosclerosis and components of metabolic syndrome (MetS) (34–36). Our study also observed that plasma levels of APOH, APOC2 and APOC3 were significantly higher in lean individuals with NAFLD compared to those without, indicating a potential association between lean NAFLD with metabolic imbalance.

Along the same lines, when comparing lean NAFLD with overweight NALFD, increased levels of APOC2, APOC3, APOC4 and CETP were found in the former. This observation aligns with previous studies suggesting that lean NAFLD individuals tend to exhibit higher visceral adiposity index scores, which is an indicator of visceral fat function associated with cardio metabolic risk, than those who are overweight or obese.

#### 3.2.5 Proteins involved in complement system and immune regulation in lean NAFLD

The complement system, a vital component of innate immunity, is predominantly produced in the liver, appears to be dysregulated in lean NAFLD patients. Specifically, the levels of C8B, C9, C4BPA, CFH, CFP, IGHV3-7, and THBS1, which are involved in complement system and immune regulation, were found to be significantly differently expressed in lean NAFLD group compared to lean healthy controls. C8B, C9, C4BPA and CFH were of high diagnosis value of lean NAFLD (AUC > 0.8). These results may indicate liver damage in lean NAFLD patients. Notably, the lean NAFLD group exhibited significantly elevated levels of plasma CFH, which has been implicated in insulin resistance and the pathophysiology of various inflammation-mediated diseases.

Other proteins related to immune and complement system, such as inter-α-trypsin inhibitor that has been identified as complement factors-interacting molecule and inhibit complement activation through the classical and alternative pathways, were also identified as potential biomarkers for lean NAFLD (37).

#### 3.2.6 Plasma proteins highly associated with lean NAFLD

Platelet activation is known to increase in patients with MetS and obesity, likely reflecting the alterations in the platelet membrane component. The plasma proteomics data showed that 7 of 8 DEPs involved in platelet degranulation (APOH, CAP1, F13A1, ITIH3, QSOX1, SPP2, THBS1, and VWF) were downregulated in lean NAFLD patients. Among these, von Willebrand factor (VWF), which is known to mediate platelet adhesion and aggregation and is elevated in NAFLD patients, however, was significantly decreased in the lean NAFLD group.

A significant association was also found between lean NAFLD and several plasma proteins linked to liver injury and MetS, including afamin (AFM), insulin-like growth factor binding protein (IGFBP), gelsolin, and hepatocyte growth factor activator (HGFAC). In NAFLD, IGFBP3 levels are believed to be reduced, while elevated IGFBP3 levels correlate with atherosclerosis (38). In this study, plasma level of IGFBP3 was significantly higher in lean NAFLD and performs well as a potential biomarker (AUC > 0.7). AFM has already been proposed as potential markers for NAFLD, with a closer association to hepatic lipid accumulation, liver damage, and insulin resistance than to obesity (39). This aligns with our observation, that the lean NAFLD group had notably higher levels of AFM. The plasma levels of gelsolin were found significantly decreased in lean NAFLD patients may suggest secondary inflammation and liver injury. On the other hand, lean NAFLD patients had obviously lower levels of HGFAC than lean healthy individuals, which should retard repair of damaged livers.

Matrix metalloproteinases (MMPs) involved in the turnover of fibrosis were also differently expressed in lean NAFLD patients. In comparison to the lean control group, we observed increased plasma levels of MMP9 but decreased levels of MMP2 in the lean NAFLD group. Coagulation factors would be expected to decrease when liver functions are impaired (40), even though in lean NAFLD patients, we only observed reductions in plasma coagulation factor XIII A chain (F13A1) levels.

## 4 Discussion

Within a metabolic continuum there is a normal weight classification of metabolically obese, defined as lean individuals who present with insulin resistance, hyperinsulinemia and atherogenic dyslipidaemia. Asians have been shown to develop significant metabolic disease outcomes at a lower BMI than other ethnic groups. The reason is not entirely clear. However, several factors have been proposed, including differences in body composition, genetics, and environmental factors such as diet and physical activity. As NAFLD is believed to be a hepatic manifestation of MetS, it is not surprising that the incidence of lean NAFLD in Chinese populations is significantly higher than in Western populations for a given BMI value. On the other, in a clinical retrospective study where the relationship between NAFLD and BMI as a risk factor was investigated, the study revealed that the occurrence of NAFLD is linked to lipid deposition, rather than BMI (41).

Despite the generally better histological and biochemical profile of lean NAFLD patients compared with NAFLD patients and higher BMI, clearly, lean NAFLD is a neglected and underappreciated subtype. In fact, compared with obese individuals, NAFLD in lean populations that do not have the readily recognizable phenotype of obesity are at increased risk for future severe liver disease. It is suggested that lean individuals with NAFLD can develop advanced liver disease, metabolic comorbidities, cardiovascular disease, and even mortality related to the liver. Despite lower fibrosis stages, the lean cohort had a higher risk of severe liver disease compared to overweight or obese NAFLD patients (10). In this context, early diagnosis of lean NAFLD is of great clinical significance. In this work, we aim to investigate the clinical and proteomic profiles of lean NAFLD. Our four study groups, namely lean NAFLD, lean control, overweight NAFLD, and overweight NAFLD, were defined based on BMI, as this is the most used tool for assessing normal and abnormal weight as part of the clinical routine.

We first cross-compared the clinical characteristics of the four groups. Both the lean and overweight NAFLD groups had slightly higher BMIs than their respective healthy counterparts. Despite all clinical parameters of the enrolled NAFLD patients being within normal ranges, we found that lean NAFLD patients were more likely to have components of the MetS and worse liver function tests compared to lean controls. Notably, lean NAFLD patients had significantly higher levels of liver enzymes, such as ALT and AST. Moreover, we observed that lean NAFLD patients had high LDL-C, hypertriglyceridemia, and higher levels of fasting blood glucose. Several blood indicators, including RBC, WBC, HGB, and HCT, were also higher in lean NAFLD patients. Although metabolic disorders may not directly affect blood cell counts, a complete blood cell count test can provide essential clinical information about the patient’s overall health status and the presence of any underlying medical conditions. Research has shown that RBC and WBC counts are associated with MetS and insulin resistance. Furthermore, elevated levels of HGB and HCT values are often seen in overweight patients with predominant insulin resistance, and people with a raised HGB level are at a higher risk of developing abnormal liver function (42). Another study has suggested that WBC count is related to the occurrence of NAFLD (43).

Consistent with other reports, we also found that for the aforementioned clinical variables, lean NAFLD patients were in between overweight NAFLD and lean healthy controls, suggesting that lean NAFLD subjects may have favorable metabolic and pathological profiles than overweight NAFLD subjects. Of note, our lean NAFLD subjects had significant higher level of TSH than overweight NAFLD. TSH is an important hormone that regulates metabolism, including lipid metabolism in the liver. Higher levels of TSH within the normal range have also been linked to dyslipidemia. A large cross-sectional study involving 20,783 subjects in Spain demonstrated that TSH levels were positively associated with total cholesterol (TC) and low-density lipoprotein cholesterol (LDL-C) levels and negatively associated with high-density lipoprotein cholesterol (HDL-C) levels (23). Recent systematic reviews and meta-analyses suggested that, elevated TSH levels may be associated with the development and progression of NAFLD (44). Although previous studies on the relationship between thyroid function and NAFLD risk have been inconsistent and controversial, our clinical data poorer thyroid function may be closely related to dyslipidemia in lean individuals with NAFLD. Additional prospective research is needed to address these underlying mechanisms of thyroid function in lean NAFLD.

NAFLD has been consistently associated with a higher prevalence of chronic kidney disease (CKD) in numerous observational studies, indicating that individuals with NAFLD are at a significantly greater risk of developing CKD compared to those without the condition (24–26). Uric acid (UA), the end product of the breakdown of unwanted purines in humans, has been associated with a number of metabolic disorders. Consistent with our data, Zheng et al. demonstrated positive associations between high serum UA concentrations and the risk of lean NAFLD in Chinese adults, independent of the other metabolic factors (45). Based on these findings, serum UA could be considered as a simple and non-invasive marker for follow-up of patients with lean-NAFLD. In an earlier study in which the diagnosis of NAFLD was based on blood tests, ultrasound imaging, and the liver/spleen ratio of computed tomography values, they showed that patients with NAFLD had significantly higher levels of BUN compared with control subjects. Creatinine has been reported as a factor associated with NAFLD in several studies (37), whereas we did not observe statistically significant differences between NAFLD patients and controls in both overweight and lean cohorts.

Platelets are well-known to play a role in the vascular complications of MetS and atherosclerosis, and emerging evidence suggests that they may also be involved in NAFLD. Many studies have investigated the relationship between platelets and NAFLD, with findings suggesting that NAFLD patients often have an elevated mean platelet volume (MPV), which is a reliable indicator of platelet activation (21). Some studies have reported lower platelet counts and higher MPV in NAFLD patients, nonetheless other researchers did not confirm these alterations (46, 47). In contrast to the above studies, our comparison of overweight NAFLD patients and overweight controls revealed that NAFLD patients had significantly higher platelet counts and significantly lower MPV. In the lean cohort, there was no significant difference in either MPV or platelet count between NAFLD patients and controls. In our large-scale samples, we observed significant lower platelet counts only in the comparison of lean NAFLD patients to lean controls. However, we found no statistical difference in MPV in both lean and obese NAFLD patients, despite previous reports that it is directly correlated with histological severity of hepatic inflammation and fibrosis.

These clinical findings underscore the importance of investigating NAFLD in lean subjects. On the other hand, currently established non-invasive methods in clinical practice for the diagnosis and NAFLD prognosis has some limitations; for example, it may not be sufficiently sensitive in the early stages of the disease, especially for lean subjects. To address this gap, mass spectrometry-based proteomics technology holds great potential in gain novel insights into disease mechanism and discovering new biomarkers. Therefore, we conducted a discovery proteomics analysis on 20 samples from each study group, with the aim of identifying novel proteins associated with lean NAFLD and understanding plasma protein changes in this condition among lean subjects. Based on pairwise comparisons of the proteomic data, we found that lean NAFLD and overweight NAFLD exhibit distinct proteomic profiles, and the two NAFLD entities may have different pathogenesis. We identified 62 DEPs that could predict the occurrence of NAFLD in lean subjects, which may provide a rich biomarker pool for lean NAFLD diagnosis. A considerable proportion of the DEPs are liver-specific or liver-enriched.

Apolipoproteins are structurally and functionally important lipid-transporting proteins in the blood circulation. As NAFLD bears strong associations with insulin resistance and dyslipidemia, we would expect plasma apolipoprotein concentrations to be altered in patients with chronic liver disease. APOF and APOH were found to have significant diagnostic value for lean NAFLD. APOF concentrations are considerably higher in individuals with high cholesterol levels but lower in those with high triglyceride levels. It has been notes that the response of APOF to plasma triglyceride levels is sex-varied. In male with high triglyceride levels, APOF levels are approximately half that of normolipidemic plasma, whereas in females, APOF levels have an upward trend. Despite The relationship between APOF and blood lipids remains controversial, nearly all of the reported data support the relationship between APOH and lipid metabolism, thrombosis, and inflammation. Increased levels of APOH were also associated with the presence of clinically evident components of arteriosclerosis and MetS (34). Previous studies have reported that serum levels of APOC2 and APOC3 were significantly higher in patients with MetS compared to those without (35, 36). We found only NAFLD patients within the lean cohort showed dysregulated apolipoproteins such as APOH, APOB, APOC2, and APOC3, implying that their lipid metabolism problems may differ from those of overweight or obese populations.

An important arm of the immune system is the complement cascade, which is controlled by a balance of activator and regulator proteins (48). Over-activation or dysregulation of the complement system can have far reaching clinical consequences. The complement system has been shown to be involved in NAFLD progression (49). The mechanisms of complement activation and regulation within the liver are incompletely understood. A considerable number of the significantly changed proteins in lean NAFLD patients are associated with complement and immune system. Among these, CFH has been implicated in insulin resistance, as well as pathophysiology of various inflammation-mediated diseases. It is reported that increased circulating CFH concentrations were observed in subjects with altered glucose tolerance, which could reflect the decreased insulin sensitivity and metabolic disturbances (50). Given that insulin resistance is an independent risk factor for NAFLD, it is not surprising that the lean NAFLD group exhibited significantly elevated levels of plasma CFH.

Platelets play a pivotal role in both hepatic homeostasis and the liver’s response to injury. As mentioned above, in lean cohort, no significant differences in platelet count were observed between NAFLD patients and their healthy counterparts. However, the plasma proteomics data showed that 7 of 8 DEPs involved in platelet degranulation were downregulated in lean NAFLD patients. Of these, von Willebrand factor (VWF), a mediator of platelet adhesion and aggregation that has been shown to be elevated in patients with NAFLD, however, was found to be significantly decreased in the lean NAFLD group.

In addition to above proteins, we also observed a significant association between lean NAFLD and certain plasma proteins related to liver injury and MetS, represented by AFM, IGFBP, gelsolin, and HGFAC. The role of IGFBP3 in NAFLD is multifaceted. IGFBP3 is the major insulin binding protein such as growth factor 1 (IGF1) which is a stimulator of the production of IGFBP3. IGF1 is secreted by hepatocytes under growth hormone stimulation and has been shown to be protective in ischemic heart disease as well as in atherosclerosis. In NAFLD, IGFBP3 levels are believed to be reduced, while elevated IGFBP3 levels correlate with atherosclerosis (38). In this study, plasma level of IGFBP3 was significantly higher in lean NAFLD and performs well as a potential biomarker (AUC > 0.7). AFM is predominantly expressed in liver and secreted into circulation. The Studies published to date demonstrate an increased AFM rate in patients with components of the MetS (51, 52), NAFLD (53), and alcoholic liver disease (ALD). It has previously been suggested that AFM may be a marker for NAFLD, since AFM was more closely linked to hepatic lipid accumulation, hepatic injury and insulin resistance than obesity (39). This is consistent with our observation that significantly higher AFM levels were found in the lean NAFLD group. Plasma gelsolin (GSN) has multiple physiological functions, such as being a substrate for extracellular matrix modulating enzymes, participating in the extracellular actin sensor system, and presenting inflammatory mediators to their receptors (54, 55). Consequently, gelsolin levels significantly may decrease after tissue injury in various conditions, including acute respiratory distress syndrome, acute injury to the lungs and liver, sepsis, major trauma, prolonged hyperoxia, and malaria (56, 57). Gelsolin could not be considered as a specific marker of lean NAFLD, however, the significantly decreased levels of plasma gelsolin in lean NAFLD patients may suggest secondary inflammation and liver injury. The growth factor activator Hepatocyte (HGFAC) is a primary activator of proHGF (the precursor form of hepatocyte growth factor) at the site of tissue damage, promoting accelerated healing of injured tissue, HGFAC deficiency can significantly disrupt subsequent tissue regeneration and repair (58). HGFAC is mainly synthesized by hepatocytes and circulates in the plasma. Lean NAFLD patients had obviously lower levels of HGFAC than lean healthy individuals, which should attenuated proHFG activation, thereby retarding repair of damaged livers.

## 5 Conclusions

Previous studies have shown several distinct proteins or patterns that differentiate end-stage liver diseases, particularly hepatocellular carcinoma. However, early-stage NAFLD may be overlooked. On the other hand, lean-type NAFLD, which lacks the typical obesity phenotype, is usually asymptomatic and may not seek medical advice. Due to the progressive nature of NAFLD, the early diagnosis and early intervention of lean NAFLD is of great clinical interest.

In this work, the large-scale clinical data revealed individuals with NAFLD who are lean have a distinct clinical profile from those who are overweight. Lean NAFLD patients exhibit worse metabolic profiles compared to their healthy counterparts, but generally experience fewer systemic metabolic issues than NAFLD subjects who are additionally obese.

By mass spectrometry-based proteomics technology, we were able to identify dozens of differentially expressed plasma proteins that could predict the occurrence of NAFLD in lean subjects, which may provide a novel biomarker pool for lean NAFLD diagnosis. The observed protein alterations in lean NAFLD indicate changes in lipid metabolism and inflammatory processes and complement activation, processes known to be associated with NAFLD. To the best of our knowledge, this study represents the first investigation into plasma proteomics of lean patients suffering from NAFLD.

Even though informative, our study also has limitations that need to be addressed. To validate the diagnostic biomarkers, a targeted mass spectrometry approach should be performed on a separate cohort for further refinement. While BMI is commonly used as a surrogate for body fat content, its utility in determining true body composition, especially in the lean population, may be insufficient. It is essential to investigate the importance of body fat distribution and specific genetic polymorphisms associated with a lean NAFLD, such as PNPLA3 and TM6SF2. Moreover, in this study, NAFLD diagnosis relied on ultrasonography, which can only estimate the prevalence of the disease, but not disease severity. Therefore, efforts should also made to explore effective biomarkers for assessing the progressive stages of lean NAFLD.

## Data availability statement

The datasets presented in this study can be found in online repositories. The names of the repository/repositories and accession number(s) can be found in the article/**Supplementary Material**.

## Ethics statement

The studies involving human participants were reviewed and approved by Clinical Ethics Committee of Putuo hospital affiliated to Shanghai University of Traditional Chinese Medicine. The patients/participants provided their written informed consent to participate in this study.

## Author contributions

YJ and CH performed experiments. JZ analyzed data. ML, SD, JT and YY collected clinical serum sample. XZ prepared and wrote the manuscript. GJ reviewed the manuscript. All authors reviewed and approved the manuscript.

## Supplementary material

Supplementary Figure 1 GO enrichment of differentially expressed proteins associated with overweight NAFLD in term of biological process.

Supplementary Figure 2 Receiver operating characteristic (ROC) curves for potential diagnostic markers of overweight NAFLD with AUC values above 0.8.

Supplementary Table 1 Demographic, clinical, anthropometrical and laboratory characteristics of 80 subjects in proteomics study.

## References

[1] Lonardo A Nascimbeni F Maurantonio M Marrazzo A Rinaldi L Adinolfi LE. Nonalcoholic fatty liver disease: Evolving paradigms. World J Gastroenterol (2017) 23(36):6571–92. doi: 10.3748/wjg.v23.i36.6571 PMC5643282 29085206

[2] Ye Q Zou B Yeo YH Li J Huang DQ Wu Y. Global prevalence, incidence, and outcomes of non-obese or lean non-alcoholic fatty liver disease: a systematic review and meta-analysis. Lancet Gastroenterol Hepatol (2020) 5(8):739–52. doi: 10.1016/S2468-1253(20)30077-7 32413340

[3] Kim D Kim WR. Nonobese fatty liver disease. Clin Gastroenterol Hepatol (2017) 15(4):474–85. doi: 10.1016/j.cgh.2016.08.028 27581063

[4] Younes R Bugianesi E. NASH in lean individuals. Semin Liver Dis (2019) 39(1):86–95. doi: 10.1055/s-0038-1677517 30654392

[5] Petersen KF Dufour S Feng J Befroy D Dziura J Dalla Man C. Increased prevalence of insulin resistance and nonalcoholic fatty liver disease in Asian-Indian men. Proc Natl Acad Sci USA (2006) 103(48):18273–7. doi: 10.1073/pnas.0608537103 PMC1693873 17114290

[6] Kumar R Mohan S. Non-alcoholic fatty liver disease in lean subjects: Characteristics and implications. J Clin Transl Hepatol (2017) 5(3):216–23. doi: 10.14218/JCTH.2016.00068 PMC5606968 28936403

[7] Ahadi M Molooghi K Masoudifar N Namdar AB Vossoughinia H Farzanehfar M. A review of non-alcoholic fatty liver disease in non-obese and lean individuals. J Gastroenterol Hepatol (Australia) (2021) 36(6):1497–507. doi: 10.1111/jgh.15353 33217052

[8] Leung JCF Loong TCW Wei JL Wong GLH Chan AWH Choi PCL. Histological severity and clinical outcomes of nonalcoholic fatty liver disease in nonobese patients. Hepatology (2017) 65(1):54–64. doi: 10.1002/hep.28697 27339817

[9] Hae JK Hyeong JK Kwang EL Dae JK Soo KK Chul WA. Metabolic significance of nonalcoholic fatty liver disease in nonobese, nondiabetic adults. Arch Intern Med (2004) 164(19):2169–75. doi: 10.1001/archinte.164.19.2169 15505132

[10] Hagström H Nasr P Ekstedt M Hammar U Stål P Hultcrantz R. Risk for development of severe liver disease in lean patients with nonalcoholic fatty liver disease: A long-term follow-up study. Hepatol Commun (2018) 2(1):48–57. doi: 10.1002/hep4.1124 29404512 PMC5776871

[11] Rinella ME. Nonalcoholic fatty liver disease a systematic review. JAMA - J Am Med Assoc (2015) 313(22):2263–73. doi: 10.1001/jama.2015.5370 26057287

[12] Vilar-Gomez E Chalasani N. Non-invasive assessment of non-alcoholic fatty liver disease: Clinical prediction rules and blood-based biomarkers. J Hepatol (2018) 68(2):305–15. doi: 10.1016/j.jhep.2017.11.013 29154965

[13] Geyer PE Holdt LM Teupser D Mann M. Revisiting biomarker discovery by plasma proteomics. Mol Syst Biol (2017) 13(9):942. doi: 10.15252/msb.20156297 28951502 PMC5615924

[14] Keshishian H Burgess MW Specht H Wallace L Clauser KR Gillette MA. Quantitative, multiplexed workflow for deep analysis of human blood plasma and biomarker discovery by mass spectrometry. Nat Protoc (2017) 12(8):1683–701. doi: 10.1038/nprot.2017.054 PMC6057147 28749931

[15] Niu L Geyer PE Wewer Albrechtsen NJ Gluud LL Santos A Doll S. Plasma proteome profiling discovers novel proteins associated with non-alcoholic fatty liver disease. Mol Syst Biol (2019) 15(3):1–16. doi: 10.15252/msb.20188793 PMC6396370 30824564

[16] Fan JG Wei L Zhuang H Cai W Feng Chen D Duan ZP. Guidelines of prevention and treatment of nonalcoholic fatty liver disease (2018, China). J Dig Dis (2019) 20:163–73. doi: 10.1111/1751-2980.12685 30444584

[17] Sugiyama A Kurisu A Bunthen E Ouoba S Ko K Rakhimov A. Distribution of FIB-4 index in the general population: analysis of 75,666 residents who underwent health checkups. BMC Gastroenterol (2022) 22(1):241. doi: 10.1186/s12876-022-02290-1 35562658 PMC9101936

[18] Shah AG Lydecker A Murray K Tetri BN Contos MJ Sanyal AJ. Comparison of noninvasive markers of fibrosis in patients with nonalcoholic fatty liver disease. Clin Gastroenterol Hepatol (2009) 7(10):1104–12. doi: 10.1016/j.cgh.2009.05.033 PMC3079239 19523535

[19] Tokushige K Ikejima K Ono M Eguchi Y Kamada Y Itoh Y. Evidence-based clinical practice guidelines for nonalcoholic fatty liver disease/nonalcoholic steatohepatitis 2020. J Gastroenterol (2021) 56(11):951–63. doi: 10.1007/s00535-021-01796-x PMC8531062 34533632

[20] Wiśniewski JR Zougman A Nagaraj N Mann M. Universal sample preparation method for proteome analysis. Nat Methods (2009) 6(5):359–62. doi: 10.1038/nmeth.1322 19377485

[21] Chauhan A Adams DH Watson SP Lalor PF. Platelets: No longer bystanders in liver disease. Hepatology (2016) 64(5):1774–84. doi: 10.1002/hep.28526 PMC5082495 26934463

[22] Bano A Chaker L Plompen EPC Hofman A Dehghan A Franco OH. Thyroid function and the risk of nonalcoholic fatty liver disease: The Rotterdam study. J Clin Endocrinol Metab (2016) 101(8):3204–11. doi: 10.1210/jc.2016-1300 27270473

[23] Santos-Palacios S Brugos-Larumbe A Guillén-Grima F Galofré JC. A cross-sectional study of the association between circulating TSH level and lipid profile in a large Spanish population. Clin Endocrinol (Oxf) (2013) 79(6):874–81. doi: 10.1111/cen.12216 23550997

[24] Marcuccilli M Chonchol M. NAFLD and chronic kidney disease. Int J Mol Sci (2016) 17(4):562. doi: 10.3390/ijms17040562 27089331 PMC4849018

[25] Targher G Chonchol MB Byrne CD. CKD and nonalcoholic fatty liver disease. Am J Kidney Dis (2014) 64(4):638–52. doi: 10.1053/j.ajkd.2014.05.019 25085644

[26] Sesti G Fiorentino TV Arturi F Perticone M Sciacqua A Perticone F. Association between noninvasive fibrosis markers and chronic kidney disease among adults with nonalcoholic fatty liver disease. PloS One (2014) 9(2):e88569. doi: 10.1371/journal.pone.0088569 24520400 PMC3919760

[27] Becker J Friedman E. Renal function status. Am J Roentgenol (2013) 200:827–9. doi: 10.2214/AJR.12.9872 23521456

[28] Saki F Karamizadeh Z Honar N Moravej H Ashkani-Esfahani S Namvar Shooshtarian MH. Association of plasma retinol binding protein-4 (RBP4) and sonographic grading of fatty liver in obese Iranian children. Hepat Mon (2012) 12(12):e7103. doi: 10.5812/hepatmon.7103 23423766 PMC3575548

[29] Yang Q Graham TE Mody N Preitner F Peroni OD Zabolotny JM. Serum retinol binding protein 4 contributes to insulin resistance in obesity and type 2 diabetes. Nature (2005) 436(7049):356–62. doi: 10.1038/nature03711 16034410

[30] Małecki P Tracz J Łuczak M Figlerowicz M Mazur-Melewska K Służewski W. Serum proteome assessment in nonalcoholic fatty liver disease in children: a preliminary study. Expert Rev Proteomics (2020) 17(7-8):623–32. doi: 10.1080/14789450.2020.1810020 32921203

[31] Cole BK Feaver RE Wamhoff BR Dash A. Non-alcoholic fatty liver disease (NAFLD) models in drug discovery. Expert Opin Drug Discovery (2018) 13(2):193–205. doi: 10.1080/17460441.2018.1410135 29190166

[32] Lim JW Dillon J Miller M. Proteomic and genomic studies of non-alcoholic fatty liver disease - Clues in the pathogenesis. World J Gastroenterol (2014) 20(26):8325–40. doi: 10.3748/wjg.v20.i26.8325 PMC4093687 25024592

[33] Liu Y Morton RE. Apolipoprotein F: a natural inhibitor of cholesteryl ester transfer protein and a key regulator of lipoprotein metabolism. Curr Opin Lipidol (2020) 31(4):194–9. doi: 10.1097/MOL.0000000000000688 PMC8020876 32520778

[34] Castro A Lázaro I Selva DM Céspedes E Girona J NúriaPlana. APOH is increased in the plasma and liver of type 2 diabetic patients with metabolic syndrome. Atherosclerosis (2010) 209(1):201–5. doi: 10.1016/j.atherosclerosis.2009.09.072 19878946

[35] Sumida Y Nakajima A Itoh Y. Limitations of liver biopsy and non-invasive diagnostic tests for the diagnosis of nonalcoholic fatty liver disease/nonalcoholic steatohepatitis. World J Gastroenterol (2014) 20(2):475–85. doi: 10.3748/wjg.v20.i2.475 PMC3923022 24574716

[36] Boiko AS Mednova IA Kornetova EG Semke AV Bokhan NA Loonen AJM. Apolipoprotein serum levels related to metabolic syndrome in patients with schizophrenia. Heliyon (2019) 5(7):e02033. doi: 10.1016/j.heliyon.2019.e02033 31317083 PMC6611937

[37] Garantziotis S Hollingsworth JW Ghanayem RB Timberlake S Zhuo L Kimata K. A. Inter-α-trypsin inhibitor attenuates complement activation and complement-induced lung injury. J Immunol (2007) 179(6):4187–92. doi: 10.4049/jimmunol.179.6.4187 17785858

[38] Miller MH Walsh SV Atrih A Huang JTJ Ferguson MAJ Dillon JF. The serum proteome of nonalcoholic fatty liver disease: A multimodal approach to discovery of biomarkers of nonalcoholic steatohepatitis. J Gastroenterol Hepatol (Australia) (2014) 29(10):1839–47. doi: 10.1111/jgh.12614 24750217

[39] Kurdiova T Balaz M Kovanicova Z Zemkova E Kuzma M Belan V. Serum afamin a novel marker of increased hepatic lipid content. Front Endocrinol (Lausanne) (2021) 12:670425. doi: 10.3389/fendo.2021.670425 34603196 PMC8481912

[40] Dickneite G Herwald H Korte W Allanore Y Denton CP Cerinic MM. Coagulation factor XIII: A multifunctional transglutaminase with clinical potential in a range of conditions. Thromb Haemost (2015) 113(4):686–97. doi: 10.1160/TH14-07-0625 25652913

[41] Ciardullo S Oltolini A Cannistraci R Muraca E Perseghin G. Sex-related association of nonalcoholic fatty liver disease and liver fibrosis with body fat distribution in the general US population. Am J Clin Nutr (2022) 115(6):1528–34. doi: 10.1093/ajcn/nqac059 35244676

[42] Bernhardt P Kratzer W Schmidberger J Graeter T Gruener B Adler G. Laboratory parameters in lean NAFLD: Comparison of subjects with lean NAFLD with obese subjects without hepatic steatosis. BMC Res Notes (2018) 11(1):1–8. doi: 10.1186/s13104-018-3212-1 29409538 PMC5801753

[43] Jiang Y Zeng J Chen B. Hemoglobin combined with triglyceride and ferritin in predicting non-alcoholic fatty liver. J Gastroenterol Hepatol (Australia) (2014) 29(7):1508–14. doi: 10.1111/jgh.12580 24628002

[44] Guo Z Li M Han B Qi X. Association of non-alcoholic fatty liver disease with thyroid function: A systematic review and meta-analysis. Dig Liver Dis (2018) 50(11):1153–62. doi: 10.1016/j.dld.2018.08.012 30224316

[45] Zheng X Gong L Luo R Chen H Peng B Ren W. Serum uric acid and non-alcoholic fatty liver disease in non-obesity Chinese adults. Lipids Health Dis (2017) 16(1):1–7. doi: 10.1186/s12944-017-0531-5 29037239 PMC5644248

[46] Ozhan H Aydin M Yazici M Yazgan O Basar C Gungor A. Mean platelet volume in patients with non-alcoholic fatty liver disease. Platelets (2010) 21(1):29–32. doi: 10.3109/09537100903391023 19947902

[47] Dalbeni A Castelli M Zoncapè M Minuz P Sacerdoti D. Platelets in non-alcoholic fatty liver disease. Front Pharmacol (2022) 13:842636. doi: 10.3389/fphar.2022.842636 35250588 PMC8895200

[48] Zipfel PF Skerka C. Complement regulators and inhibitory proteins. Nat Rev Immunol (2009) 9(10):729–40. doi: 10.1038/nri2620 19730437

[49] Laskowski J Renner B Pickering MC Serkova NJ Smith-Jones PM Clambey ET. Complement factor H–deficient mice develop spontaneous hepatic tumors. J Clin Invest (2020) 140(8):4039–54. doi: 10.1172/JCI135105 PMC7410061 32369457

[50] Moreno-Navarrete JM Martínez-Barricarte R Catalán V Sabater M Gómez-Ambrosi J Ortega FJ. Complement factor H is expressed in adipose tissue in association with insulin resistance. Diabetes (2010) 59(1):200–9. doi: 10.2337/db09-0700 PMC2797922 19833879

[51] Dieplinger H Dieplinger B. Afamin–A pleiotropic glycoprotein involved in various disease states. Clin Chim Acta (2015) 446:105–10. doi: 10.1016/j.cca.2015.04.010 25892677

[52] Kurdiova T Balaz M Kovanicova Z Zemkova E Kuzma M Belan V. Serum afamin a novel marker of increased hepatic lipid content. Front Endocrinol (Lausanne) (2021) 12:670425(September). doi: 10.3389/fendo.2021.670425 34603196 PMC8481912

[53] Bell LN Theodorakis JL Vuppalanchi R Saxena R Bemis KG Wang M. Serum proteomics and biomarker discovery across the spectrum of nonalcoholic fatty liver disease. Hepatology (2010) 51(1):111–20. doi: 10.1002/hep.23271 PMC2903216 19885878

[54] Bucki R Levental I Kulakowska A Janmey P. Plasma gelsolin: function, prognostic value, and potential therapeutic use. Curr Protein Pept Sci (2008) 9(6):541–51. doi: 10.2174/138920308786733912 19075745

[55] Marrocco C Rinalducci S Mohamadkhani A D’Amici GM Zolla L. Plasma gelsolin protein: A candidate biomarker for hepatitis B-associated liver cirrhosis identified by proteomic approach. Blood Transfusion (2010) 8(Suppl. 3):s105–112. doi: 10.2450/2010.017S PMC2897206 20606740

[56] Piktel E Levental I Durnaś B Janmey PA Bucki R. Plasma gelsolin: Indicator of inflammation and its potential as a diagnostic tool and therapeutic target. Int J Mol Sci (2018) 19(9):2516. doi: 10.3390/ijms19092516 30149613 PMC6164782

[57] Bhosale SD Moulder R Venäläinen MS Koskinen JS Pitkänen N Juonala MT. Serum proteomic profiling to identify biomarkers of premature carotid atherosclerosis. Sci Rep (2018) 8(1):1–9. doi: 10.1038/s41598-018-27265-9 29907817 PMC6003912

[58] Fukushima T Uchiyama S Tanaka H Kataoka H. Hepatocyte growth factor activator: A proteinase linking tissue injury with repair. Int J Mol Sci (2018) 19(11):1–11. doi: 10.3390/ijms19113435 PMC6275078 30388869

