# Supplementary figures and images for: Clinical characterization and proteomic profiling of lean nonalcoholic fatty liver disease
Source: Front Endocrinol (Lausanne). 2023 Nov 16;14:1171397. doi: 10.3389/fendo.2023.1171397 (PMC10687542; doi:10.3389/fendo.2023.1171397)

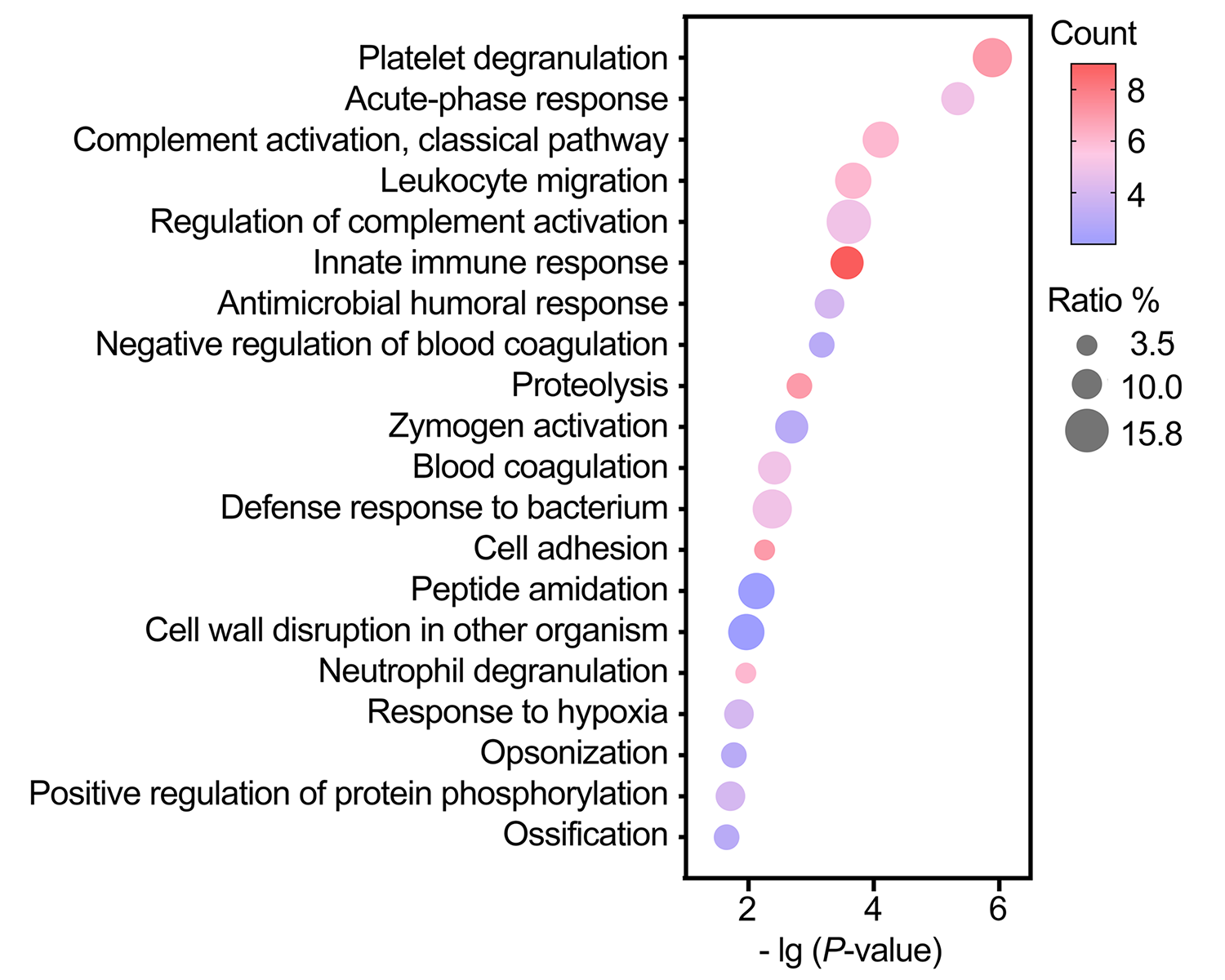

Supplement: Supplementary Figure 1 — GO enrichment of differentially expressed proteins associated with overweight NAFLD in term of biological process. [file Image_1.tif]

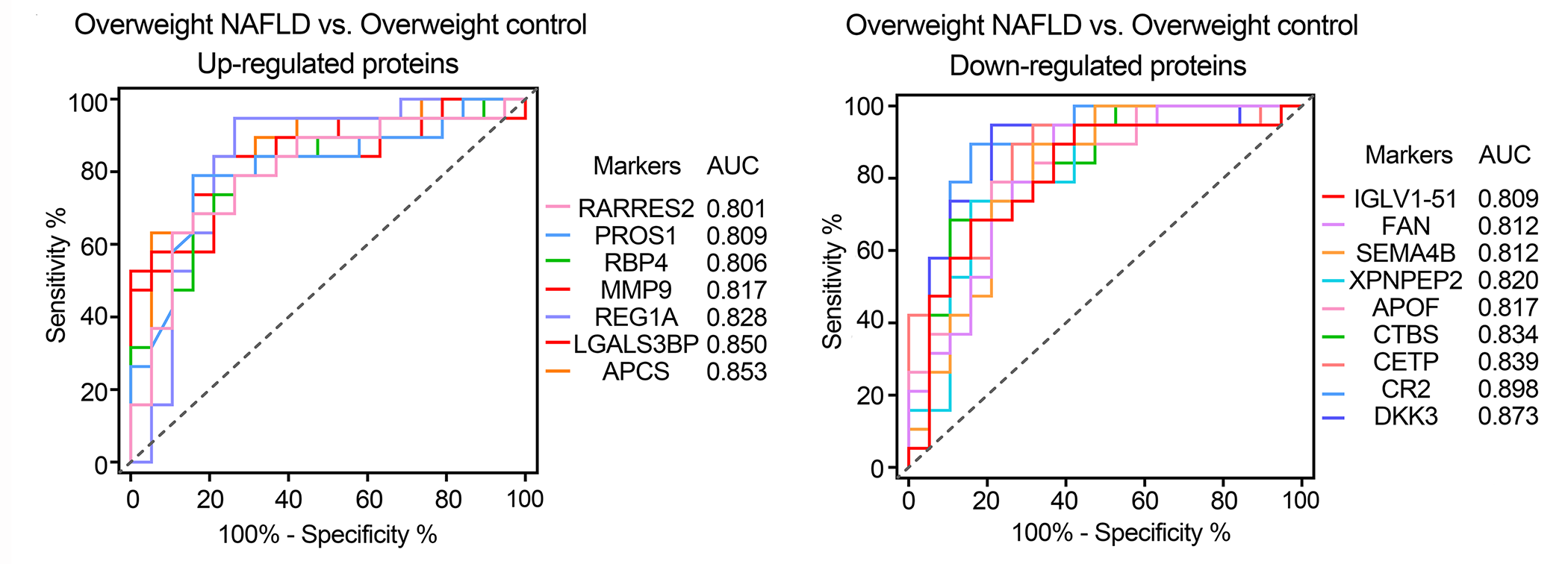

Supplement: Supplementary Figure 2 — Receiver operating characteristic (ROC) curves for potential diagnostic markers of overweight NAFLD with AUC values above 0.8. [file Image_2.tif]
