# Supplementary material for: Clinical characterization and proteomic profiling of lean nonalcoholic fatty liver disease
Source: Front Endocrinol (Lausanne). 2023 Nov 16;14:1171397. doi: 10.3389/fendo.2023.1171397 (PMC10687542; doi:10.3389/fendo.2023.1171397)
Supplement: Supplementary Table 1 — Demographic, clinical, anthropometrical and laboratory characteristics of 80 subjects in proteomics study. [file Table_1.docx]

**Table S1.** List of proteins found to be significantly differentially abundant between overweight NAFLD patients and overweight controls, with efficiency comparison of diagnostic indicators.

| Gene Symbol | Protein | P value  (ON vs. OC) | FC (ON/OC) | AUC | CI (95%) | |
| --- | --- | --- | --- | --- | --- | --- |
|  |  |  |  |  | lower | upper |
| CR2 | Complement receptor type 2 | 0.0000 | 0.38 | 0.898 | 0.794 | 1 |
| DKK3 | Dickkopf-related protein 3 | 0.0002 | 0.44 | 0.873 | 0.750 | 0.996 |
| APCS | Serum amyloid P-component | 0.0001 | 1.42 | 0.853 | 0.729 | 0.978 |
| LGALS3BP | Galectin-3-binding protein | 0.0001 | 1.49 | 0.850 | 0.722 | 0.979 |
| CETP | Cholesteryl ester transfer protein | 0.0002 | 0.48 | 0.839 | 0.708 | 0.971 |
| CTBS | Di-N-acetylchitobiase | 0.0016 | 0.58 | 0.834 | 0.703 | 0.964 |
| REG1A | Lithostathine-1-alpha | 0.0028 | 1.54 | 0.828 | 0.684 | 0.973 |
| XPNPEP2 | Xaa-Pro aminopeptidase 2 | 0.0003 | 0.45 | 0.820 | 0.682 | 0.958 |
| MMP9 | Matrix metalloproteinase-9 | 0.0030 | 2.03 | 0.817 | 0.680 | 0.954 |
| APOF | Apolipoprotein F | 0.0011 | 0.61 | 0.817 | 0.682 | 0.952 |
| SEMA4B | Semaphorin-4B | 0.0002 | 0.42 | 0.812 | 0.670 | 0.953 |
| FAH | Fumarylacetoacetase | 0.0005 | 0.39 | 0.812 | 0.673 | 0.95 |
| PROS1 | Vitamin K-dependent protein S | 0.0005 | 1.23 | 0.809 | 0.665 | 0.952 |
| IGLV1-51 | Immunoglobulin lambda variable 1-51 | 0.0021 | 0.38 | 0.809 | 0.664 | 0.954 |
| RBP4 | Retinol-binding protein 4 | 0.0008 | 1.39 | 0.806 | 0.665 | 0.947 |
| RARRES2 | Retinoic acid receptor responder protein 2 | 0.0020 | 1.68 | 0.801 | 0.655 | 0.947 |
| CD5L | CD5 antigen-like | 0.0129 | 1.27 | 0.792 | 0.633 | 0.951 |
| GP5 | Platelet glycoprotein V | 0.0033 | 0.65 | 0.792 | 0.647 | 0.938 |
| TXN | Thioredoxin | 0.0039 | 1.71 | 0.784 | 0.631 | 0.937 |
| IGHV3-7 | Immunoglobulin heavy variable 3-7 | 0.0328 | 0.83 | 0.784 | 0.623 | 0.945 |
| LCN2 | Neutrophil gelatinase-associated lipocalin | 0.0225 | 1.95 | 0.781 | 0.631 | 0.931 |
| C1RL | Complement C1r subcomponent-like protein | 0.0030 | 1.35 | 0.781 | 0.631 | 0.931 |
| CNTN4 | Contactin-4 | 0.0023 | 0.70 | 0.781 | 0.625 | 0.938 |
| COL6A1 | Collagen alpha-1(VI) chain | 0.0022 | 0.41 | 0.781 | 0.626 | 0.936 |
| PFN1 | Profilin-1 | 0.0253 | 1.77 | 0.776 | 0.622 | 0.929 |
| F9 | Coagulation factor IX | 0.0060 | 1.34 | 0.767 | 0.606 | 0.928 |
| GOLM1 | Golgi membrane protein 1 | 0.0133 | 0.58 | 0.767 | 0.609 | 0.926 |
| PAM | Peptidyl-glycine alpha-amidating monooxygenase | 0.0057 | 1.54 | 0.759 | 0.602 | 0.916 |
| SERPINA11 | Serpin A11 | 0.0198 | 0.65 | 0.756 | 0.593 | 0.92 |
| CA1 | Carbonic anhydrase | 0.0087 | 0.57 | 0.756 | 0.599 | 0.913 |
| S100A8 | Protein S100-A8 | 0.0053 | 2.10 | 0.753 | 0.590 | 0.917 |
| TKT | Transketolase | 0.0072 | 1.60 | 0.751 | 0.587 | 0.914 |
| FCN2 | Ficolin-2 | 0.0130 | 1.48 | 0.745 | 0.585 | 0.906 |
| PROC | Vitamin K-dependent protein C | 0.0109 | 1.27 | 0.745 | 0.580 | 0.91 |
| GP6 | Platelet glycoprotein VI | 0.0275 | 1.52 | 0.742 | 0.583 | 0.902 |
| THBS1 | Thrombospondin-1 | 0.0158 | 0.76 | 0.737 | 0.572 | 0.901 |
| GPR126 | Adhesion G-protein coupled receptor G6 | 0.0095 | 1.43 | 0.734 | 0.571 | 0.897 |
| ENPP2 | Ectonucleotide pyrophosphatase/phosphodiesterase family member 2 | 0.0198 | 0.69 | 0.734 | 0.570 | 0.898 |
| HSPA8 | Heat shock cognate 71 kDa protein | 0.0307 | 1.30 | 0.726 | 0.562 | 0.89 |
| SAA4 | Serum amyloid A-4 protein | 0.0260 | 0.74 | 0.726 | 0.561 | 0.891 |
| UBE2L5P | Ubiquitin-conjugating enzyme E2 L5 | 0.0393 | 1.86 | 0.715 | 0.548 | 0.881 |
| REG3A | Regenerating islet-derived protein 3-alpha | 0.0259 | 1.40 | 0.709 | 0.543 | 0.876 |
| NEO1 | Neogenin | 0.0352 | 0.51 | 0.709 | 0.538 | 0.88 |
| OAF | Out at first protein homolog | 0.0494 | 1.50 |  |  |  |
| PLEK | Pleckstrin | 0.0016 | 1.68 |  |  |  |
| CBLN4 | Cerebellin-4 | 0.0118 | 0.48 |  |  |  |
| PTGDS | Prostaglandin-H2 | 0.0184 | 1.70 |  |  |  |
| TAGLN2 | Transgelin-2 | 0.0224 | 0.69 |  |  |  |
| S100A12 | Protein S100-A12 | 0.0235 | 1.66 |  |  |  |
| AHSG | Alpha-2-HS-glycoprotein | 0.0240 | 0.81 |  |  |  |
| PGLYRP2 | N-acetylmuramoyl-L-alanine amidase | 0.0244 | 0.81 |  |  |  |
| CD34 | Hematopoietic progenitor cell antigen CD34 | 0.0255 | 0.40 |  |  |  |
| IGLL5 | Immunoglobulin lambda-like polypeptide 5 | 0.0295 | 0.37 |  |  |  |
| SBSN | Suprabasin OS=Homo sapiens | 0.0297 | 0.50 |  |  |  |
| CACNA2D1 | Voltage-dependent calcium channel subunit alpha-2/delta-1 | 0.0310 | 0.73 |  |  |  |
| FLT4 | Vascular endothelial growth factor receptor 3 | 0.0336 | 2.13 |  |  |  |
| MGP | Matrix Gla protein | 0.0369 | 1.43 |  |  |  |
| TFRC | Transferrin receptor protein 1 | 0.0449 | 0.72 |  |  |  |
| MAN2A1 | Alpha-mannosidase 2 | 0.0462 | 1.46 |  |  |  |

ON, overweight NAFLD; OC, overweight control.
