# Supplementary material for: Clinical characterization and proteomic profiling of lean nonalcoholic fatty liver disease
Source: Front Endocrinol (Lausanne). 2023 Nov 16;14:1171397. doi: 10.3389/fendo.2023.1171397 (PMC10687542; doi:10.3389/fendo.2023.1171397)
Supplement: Supplementary file 4 [file Table_2.docx]

**Table S2.** List of proteins found to be significantly differentially abundant between lean NAFLD and overweight NAFLD patients.

| Gene Symbol | Protein | P value (LN vs. ON) | FC  (LN vs. OV) |
| --- | --- | --- | --- |
| ALB | Serum albumin | 0.0371 | 1.23 |
| ACTA1 | Actin, alpha skeletal muscle | 0.0003 | 0.53 |
| APOC3 | Apolipoprotein C-III | 0.0301 | 1.59 |
| APOC4 | Apolipoprotein C-IV | 0.0144 | 1.97 |
| APOF | Apolipoprotein F | 0.0002 | 0.60 |
| ARHGDIB | Rho GDP-dissociation inhibitor 2 | 0.0017 | 0.72 |
| ATRN | Attractin | 0.0000 | 1.28 |
| C8A | Complement component C8 alpha chain | 0.0026 | 0.77 |
| C8B | Complement component C8 beta chain | 0.0003 | 0.67 |
| C9 | Complement component C9 | 0.0010 | 0.51 |
| CA2 | Carbonic anhydrase 2 | 0.0000 | 0.57 |
| CBLN4 | Cerebellin-4 | 0.0120 | 1.70 |
| CD34 | Hematopoietic progenitor cell antigen CD34 | 0.0024 | 2.94 |
| CD44 | CD44 antigen | 0.0059 | 0.57 |
| CETP | Cholesteryl ester transfer protein | 0.0058 | 1.93 |
| CR2 | Complement receptor type 2 | 0.0000 | 2.92 |
| CTBS | Di-N-acetylchitobiase | 0.0001 | 2.06 |
| CTSZ | Cathepsin Z | 0.0000 | 0.53 |
| F12 | Coagulation factor XII | 0.0013 | 1.49 |
| F13A1 | Coagulation factor XIII A chain | 0.0003 | 0.63 |
| FCGBP | IgGFc-binding protein | 0.0038 | 1.56 |
| FN1 | Fibronectin | 0.0011 | 0.80 |
| GPR126 | Adhesion G-protein coupled receptor G6 | 0.0019 | 0.63 |
| GSN | Gelsolin | 0.0000 | 0.65 |
| HGFAC | Hepatocyte growth factor activator | 0.0000 | 0.71 |
| IGF2 | Insulin-like growth factor II | 0.0044 | 1.31 |
| IGFBP3 | Insulin-like growth factor-binding protein 3 | 0.0005 | 1.44 |
| IGHD | Immunoglobulin heavy constant delta | 0.0044 | 1.99 |
| IGHV5-51 | Immunoglobulin heavy variable 5-51 | 0.0090 | 0.62 |
| IGKV4-1 | Immunoglobulin kappa variable 4-1 | 0.0020 | 0.71 |
| ITIH1 | Inter-alpha-trypsin inhibitor heavy chain H1 | 0.0005 | 0.81 |
| ITIH2 | Inter-alpha-trypsin inhibitor heavy chain H2 | 0.0002 | 0.67 |
| ITIH3 | Inter-alpha-trypsin inhibitor heavy chain H3 | 0.0006 | 0.68 |
| ITIH4 | Inter-alpha-trypsin inhibitor heavy chain H4 | 0.0002 | 0.75 |
| KIT | Mast/stem cell growth factor receptor Kit | 0.0021 | 2.06 |
| LTBP1 | Latent-transforming growth factor beta-binding protein 1 | 0.0028 | 1.41 |
| MMP2 | 72 kDa type IV collagenase | 0.0000 | 0.52 |
| OAF | Out at first protein homolog | 0.0017 | 0.58 |
| OGN | Mimecan | 0.0045 | 1.90 |
| OLFM1 | Noelin | 0.0043 | 1.40 |
| PF4 | Platelet factor 4 | 0.0059 | 1.34 |
| PLEK | Pleckstrin | 0.0016 | 0.64 |
| PODXL | Podocalyxin | 0.0001 | 1.48 |
| PPBP | Platelet basic protein | 0.0188 | 1.22 |
| QSOX1 | Sulfhydryl oxidase 1 | 0.0017 | 0.62 |
| REG1A | Lithostathine-1-alpha | 0.0026 | 0.67 |
| REG3A | Regenerating islet-derived protein 3-alpha | 0.0076 | 0.67 |
| S100A12 | Protein S100-A12 | 0.0000 | 0.28 |
| SERPIND1 | Heparin cofactor 2 | 0.0092 | 0.69 |
| SSC5D | Soluble scavenger receptor cysteine-rich domain-containing protein SSC5D | 0.0012 | 1.65 |
| TPM4 | Tropomyosin alpha-4 chain | 0.0067 | 0.53 |
| VNN1 | Pantetheinase | 0.0007 | 0.63 |
| VWF | von Willebrand factor | 0.0002 | 0.67 |
| XPNPEP2 | Xaa-Pro aminopeptidase 2 | 0.0000 | 2.32 |

LN, lean NAFLD; ON, overweight NAFLD.
